# Rationale and design of a randomized study of short-term food and cash assistance to improve adherence to antiretroviral therapy among food insecure HIV-infected adults in Tanzania

**DOI:** 10.1186/s12879-015-1186-3

**Published:** 2015-10-28

**Authors:** Sandra I. McCoy, Prosper F. Njau, Nancy L. Czaicki, Suneetha Kadiyala, Nicholas P. Jewell, William H. Dow, Nancy S. Padian

**Affiliations:** School of Public Health, University of California, Berkeley, Berkeley, CA USA; Ministry of Health and Social Welfare, Dar es Salaam, Tanzania; London School of Hygiene and Tropical Medicine, Room 305, 36 Gordon Square London WC1H 0PD, London, UK

**Keywords:** Food security, HIV infection, Adherence, Retention, Impact evaluation, Cash transfers, Food assistance

## Abstract

**Background:**

Food insecurity is an important barrier to retention in care and adherence to antiretroviral therapy (ART) among people living with HIV infection (PLHIV). However, there is a lack of rigorous evidence about how to improve food security and HIV-related clinical outcomes. To address this gap, this randomized trial will evaluate three delivery models for short-term food and nutrition support for food insecure PLHIV in Shinyanga, Tanzania: nutrition assessment and counseling (NAC) alone**,** NAC plus food assistance, and NAC plus cash transfers.

**Methods/Design:**

At three HIV care and treatment sites, 788 participants will be randomized into one of three study arms in a 3:3:1 ratio, stratified by site: NAC plus food assistance, NAC plus cash transfer, and NAC only. Eligible participants are: 1) at least 18 years of age; 2) living with HIV infection; 3) initiated ART in the past 90 days; and 4) food insecure, as measured with the Household Hunger Scale. PLHIV who are severely malnourished (body mass index (BMI) < 16 kg/m^2^) will be excluded. Participants randomized to receive food or cash transfers are eligible to receive assistance for up to six months, conditional on attending regularly scheduled visits with their HIV care provider. Participants will be followed for 12 months: the initial 6-month intervention period and then for another 6 months post-intervention. The primary outcome is ART adherence measured with the medication possession ratio. Secondary outcomes include 1) retention in care; 2) nutritional indicators including changes in food security, BMI, and weight gain; 3) viral suppression and self-reported ART adherence; and 4) participation in the labor force.

**Discussion:**

This rigorously designed trial will inform policy decisions regarding supportive strategies for food insecure PLHIV in the early stages of treatment. The study will measure outcomes immediately after the period of support ends as well as 6 months later, providing information on the duration of the interventions’ effect. The comparison of food to cash transfers will better inform policies favoring cash assistance or will provide rationale for the continued investment in food and nutrition interventions for PLHIV.

**Trial registration:**

ClinicalTrials.gov: NCT01957917.

## Background

Although early initiation of antiretroviral therapy (ART) among people living with HIV/AIDS (PLHIV) has significant clinical benefits and can virtually eliminate onward HIV transmission [1, 2], these benefits hinge on retention in care and high levels of ART adherence. However, in sub-Saharan Africa, food insecurity, poor nutrition, and poverty are pervasive threats to ART’s potential effectiveness [3–6]. Food insecurity in particular is recognized as a barrier to ART initiation, retention in care, and adherence [7, 8], thus fostering interest in the potential for food and nutrition interventions to improve the health of PLHIV.

People are considered food secure when they have adequate physical, social, and economic access to sufficient, safe and nutritious food that meets their dietary needs and food preferences for an active and healthy life [9]. Studies have highlighted several pathways through which food insecurity impedes adherence to treatment and care in the general adult population of PLHIV [5]. First, the fear of increased hunger experienced or anticipated by people on treatment as well as the real or perceived fears of exacerbated side effects in the absence of adequate nutritional intake can reduce adherence to treatment recommendations. In addition, people who are food insecure might avoid or delay HIV treatment and care because of food insecurity’s overlap with household economic status and the real or perceived costs of ART or HIV primary care (e.g., transportation). Furthermore, food insecurity may influence ART adherence and care seeking through mental health pathways related to the anxiety and stress associated with hunger [10]. However, there is a paucity of rigorous evidence to guide initiatives for PLHIV, including which assistance modalities are best to improve food security, clinical outcomes, and household economic stability [11, 12].

### Interventions to improve food security

Some have hypothesized that nutritional support in the form of food baskets or food supplements may minimize or eliminate the food insecurity’s negative effects on adherence to treatment and care. To date, there have been four quasi-experimental studies examining the effect of such programs on ART adherence [13–16]. All were conducted in sub-Saharan Africa and compared food supplementation programs to clinics or regions not receiving the programs. Of these, three [13, 14, 16] found significant improvements in treatment adherence among food recipients. However, none of the studies had an experimental design and several evaluated food rations designed for the entire household [13, 14], which are likely to be cost prohibitive and/or unsustainable in resource-constrained settings.

In addition, there is ongoing debate about whether cash transfers can achieve the same nutritional and clinical goals as food assistance [17–21]. Studies conducted among people in the general population (not limited to PLHIV) suggest that although both food and cash transfers increase food consumption, food transfers result in *greater* increases in caloric intake than cash transfers, may be preferred by beneficiaries over cash, and may be more appropriate when food is scarce and/or food markets are functioning poorly [19, 20, 22, 23]. However, cash transfers give beneficiaries freedom of choice, may be cheaper to distribute and easier to monitor, and may be more “efficient” (under the assumptions of neoclassical microeconomic theory) than food transfers [19–21, 23, 24]. The growing body of evidence suggesting that financial incentives can be used to increase adherence to ART and retention in care bolsters the idea that cash transfers have the potential to strengthen the HIV care continuum, and might be equally, if not more, cost-effective than nutritional support for some health outcomes, such as ART adherence [25, 26]. However, there are limited data from resource-constrained settings about whether targeted cash assistance programs are effective for improving the health of PLHIV, and if so, their relative effectiveness when compared to other modes of support. The only randomized study to directly compare the effect of food and cash transfers among PLHIV on ART adherence found no difference in adherence between the two support strategies after 8 months in a sample of 293 clients in Zambia, although there was no comparison arm to determine the impact of the interventions over and above the standard of care [27].

### Rationale for the current study

To address this research gap, we designed a randomized study of three delivery models for short-term food and nutrition support for PLHIV in Tanzania who recently initiated treatment: nutrition assessment and counseling (NAC) alone**,** NAC plus food assistance, and NAC plus cash transfers. Participants in the two active treatment arms receiving food or cash transfers are eligible to receive assistance for up to six months, conditional on attending regularly scheduled visits with their HIV care provider.

The study focuses on ART initiates for several reasons. First, PLHIV who initiate ART with a low body mass index (BMI) have two to six times higher mortality rates than non-malnourished patients in the first years of treatment even after controlling for disease stage, and these individuals are also more likely to be lost to follow-up [28–30]. This suggests that food availability is especially critical among ART initiates [31]. Second, short-term food or cash support may provide a bridge until the participant’s health improves sufficiently to improve work productivity and hence earnings. This helps prevent households from having to sell productive assets in order to purchase food, especially if the HIV-infected household member is unable to work at the time of treatment initiation. In South Africa, there is a sharp decline in labor force participation in the year before PLHIV start treatment, followed by a slow but nearly complete re-entry into the workforce after treatment initiation [32]. Thus, by intervening at the time of ART initiation, household labor supply and productivity may rebound faster, preventing lengthy unemployment spells and/or preventing job loss entirely [12, 33]. This may also reduce deleterious spillover effects on nutrition, education, and other dimensions of household welfare [12].

A six-month support period for ART initiates was selected to be consistent with current policy and practice and for comparability to previous research [13]. For example, the World Food Programme commonly provides short-term assistance of 6–8 months duration for malnourished and/or food insecure PLHIV [12, 34], as does the widely recognized Academic Model for Prevention and Treatment of HIV/AIDS (AMPATH) in Kenya [34, 35]. Furthermore, lengthy periods of food or cash assistance are not likely to be feasible, affordable, nor sustainable in resource-constrained settings.

For these reasons, we hypothesized that short-term support among ART initiates may be an effective response during an important window of opportunity (ART initiation), when the risk of morbidity and mortality is high, good ART adherence habits are being formed, and household welfare is threatened. Our study will measure adherence to treatment and care immediately after the period of support ends as well as 6 months later, providing information on the duration of effect (if any).

### Primary objectives

1. Compare the effectiveness of a NAC plus food assistance program for food insecure PLHIV starting ART versus a NAC plus cash transfer program on ART adherence at 6 months.
  *Hypothesis*: NAC plus cash assistance will be non-inferior to NAC plus food assistance on ART adherence.
2. Determine the effect of a nutrition assessment and counseling (NAC) program plus food or cash assistance for food insecure PLHIV starting ART versus NAC alone on ART adherence at 6 months.
  *Hypothesis*: Participants who receive NAC plus either food or cash transfers will have better adherence to ART than participants who receive NAC alone.

### Secondary objectives

The secondary objectives include evaluating the effectiveness of NAC plus food and cash transfers at 6 and 12 months of follow-up on: 1) retention in care; 2) nutritional indicators including changes in food security, BMI, and weight gain; 3) viral suppression and self-reported ART adherence; and 4) participation in the labor force.

## Methods

### Study setting and design

In Tanzania, 1,500,000 people are living with HIV infection and 399,886 are on ART [36]. The Global Hunger Index is 17.3, indicating serious levels of hunger [37]. The study is conducted in Shinyanga Region, a resource-poor region in the northwest “Lake Zone” of Tanzania where approximately 17 % of households have poor or borderline food consumption and HIV prevalence is 7.4 %, making this an ideal setting to examine how food and cash assistance may influence adherence to ART [38, 39].

We designed a 3-arm parallel group randomized trial to examine the effectiveness of short-term food and cash assistance added to the standard of care HIV services, including nutrition assessment and counseling (NAC). We include a standard-of-care comparison group that does not receive food or cash transfers. Participants will be recruited from 3 facilities,^1^ randomized to one of three study arms, and followed for 12 months: during the 6 month intervention period and then for another 6 months post-intervention. All sites participating in the study are currently trained in and implementing the President’s Emergency Plan for AIDS Relief (PEPFAR) Nutrition Assessment, Counseling, and Support (NACS) program [40].

### Inclusion and exclusion criteria

Eligible participants are: 1) at least 18 years of age; 2) living with HIV infection; 3) initiated ART in the ≤90 days; 4) food insecure, as measured with the Household Hunger Scale (score ≥2 indicating moderate to severe hunger in the household) [41, 42]; and 5) willing and able to provide written informed consent for the study. PLHIV who are severely malnourished (body mass index (BMI) < 16 kg/m^2^) are excluded from the study, as these individuals require therapeutic nutritional supplementation for nutritional recovery.

### Interventions

Participants are randomized to one of three study arms: NAC alone (comparison condition), NAC plus food assistance, and NAC plus cash transfers.

1. NAC alone (comparison condition): Participants in the comparison arm receive the standard HIV primary care services available at HIV care and treatment clinics in Tanzania, including NAC.
2. NAC plus food assistance: Participants in the NAC plus food assistance arm receive the standard of care services plus the opportunity to receive a monthly food basket for up to 6 consecutive months, conditional on attending their scheduled visits with their HIV care provider, which are typically monthly. The food basket consists of locally procured whole maize meal (12 kg), groundnuts (3 kg), and beans (3 kg). The composition of the food basket was determined with the input of experts in academic, government, and donor organizations and was selected to be applicable to other settings, not cost-prohibitive, and use foods available in the local markets. The food basket is intended to *supplement* the household’s food supply and is not intended to provide all of the required nutritional needs for all household members. Participants receive a targeted counseling message that “*this food is to help you stay healthy as you continue your HIV treatment, and you should use it to help you take your HIV medicine as your doctor has recommended.”* (A similar message is given to cash transfer recipients.) Food baskets (and cash transfers, described below) are distributed on the same two days per month for consistency.
3. NAC plus cash transfer: Participants in the NAC plus cash transfer arm receive all of the standard of care services plus the opportunity to receive a monthly cash transfer for up to 6 consecutive months, conditional on attending scheduled visits with their HIV care provider. The cash transfer is 22,500 Tanzanian Shillings (approximately $11 USD) exclusive of transaction fees and is equivalently valued to the food basket. This value was selected to prevent undue coercion to study participants and to be consistent with the Tanzania Social Action Fund (TASAF), a government-run anti-poverty program which targets “orphans, disabled, elderly, widows/widowers, and those infected or affected by HIV/AIDS*,”* among other vulnerable groups [43]. TASAF provides $6-18 monthly to households depending on the presence and number of vulnerable children and elderly members. The transfer for this study, at ~ $11, is within this range, ensuring that the amount is policy-relevant. Cash is transferred to participants via a mobile money service (i.e., M-PESA) and transaction fees are paid by the research study. In the rare case that a participant does not have a cell phone, we will provide cash directly.

#### Conditionality

Participants in the food or cash assistance arms of the study are eligible to receive transfers for up to the first 6 consecutive months of the study, conditional on attending their scheduled visit with their HIV care provider. Trained research assistants consult the medical record to verify visit attendance. (In rare instances when visits are scheduled 60 or 90 days apart by the provider, participants receive the total value of 2 or 3 monthly transfers, respectively, if they attend the scheduled visit). After a detailed review of scheduling patterns, we developed a decision rule for eligible visits: transfers can only be made once every 26 days (to ensure that no one receives a transfer more than once every 4 weeks), and the actual visit must occur within a 4 day window (+/− 4 days) from the scheduled visit to encourage adherence to the physician’s recommended monitoring plan.

### Randomization

Participants are randomized into the food, cash, and comparison arms in a 3:3:1 ratio, stratified by site, using random permuted block sizes of 7, 14, and 21. Randomization procedures were conducted at the University of California, Berkeley and the procedures and group assignments were inaccessible to the local research team. Randomization assignments were listed in opaque sealed envelopes that were sequentially numbered. After participants provided written informed consent, trained research staff at study clinics selected the next envelope in numeric order and broke the seal to reveal the randomization assignment. Due to logistical considerations, participants and investigators were not blinded to intervention assignments.

### Data collection

A comprehensive in-person interview about individual and household characteristics, food security, ART adherence, and health service utilization will be conducted at baseline, 6 and 12 months along with quantification of plasma viral load. Visit attendance, CD4 count, pharmacy pick-up data, and other clinical and prognostic markers will be abstracted from medical and pharmacy records at 0, 6, and 12 months.

### Primary outcome

The primary outcome is ART adherence at 6 months, measured with the medication possession ratio (MPR), the proportion of time an individual is in possession of >1 ART dose or prescription for ART [44]. MPR is computed from pharmacy dispensing records as the number of days ARVs are prescribed or dispensed divided by the number of days in the interval, and has been shown to be associated with short-term virologic outcomes [44–47]. We will determine the proportion of patients with MPR ≥95 % (the 95 % cutpoint is based on prior studies [48]) during the 0–6 month interval (primary outcome).

### Secondary outcomes

1. Retention in care will be measured by abstracting all scheduled and unscheduled visits from the medical record. We will use these dates to compute retention in care in several ways, following Mugavero et al.’s recommendations about how to define and measure retention in care in the absence of a gold standard [49]. We will first determine “appointment adherence,” the proportion of scheduled visits that are completed during the 0–6 and 6–12 month observation periods. We will also compute the number of “missed visits,” to facilitate comparisons to previous studies, and parameterize this indicator both as a binary and continuous variable for the analysis.
2. Change in food security, as measured by the validated Household Hunger Scale [42] and the Household Dietary Diversity Score [50].
3. Change in BMI, computed as body weight in kilograms (kg) divided by height in meters squared;
4. Weight gain (kg); and
5. Viral suppression, measured as the proportion of participants with viral load <400 copies/mL at 6 and 12 months;
6. Self-reported ART adherence, measured with a visual analog scale [51] and defined as the proportion of patients who reported taking all of their prescribed doses in the previous 5 days, averaging across all drugs in the patient's regimen, at 6 and 12 months;
7. Participation in the labor force, including whether the participant is currently working and the number of days the participant was unable to work during the study because of illness or disability.

### Sample size and power

The target sample size for Objective #1 was determined using a non-inferiority design to determine the comparative effect of NAC plus food or cash transfers on ART adherence. Given the emerging evidence demonstrating the benefits of food assistance on ART adherence [13, 14, 16], we determined that the most policy-relevant question was whether NAC plus cash assistance was *at least* as effective as NAC plus food assistance. We estimated that 75 % of participants in the food group would have a MPR ≥95 % based on data from two systematic reviews and a cohort of Tanzanian adults on ART [52–54]. We set a non-inferiority limit (∆) of 10 percentage points, such that that the cash group can have no fewer than 65 % of participants with MPR ≥95 % to be determined non-inferior. With these assumptions, power = 80 % and alpha = 0.025 for a one-sided hypothesis test, 295 participants are required in each of the cash and food transfer arms. Assuming 15 % loss to follow-up, 339 participants are required in each of the food and cash transfer arms. (For comparison, a two-sided test of superiority indicates that that the minimum detectable effect size is 9.3 percentage points with 295 participants per group, assuming 75 % of participants in the food group have a MPR ≥95 %).

To determine whether NAC plus food or cash transfers is better than NAC alone (Objective #2), we require a comparison group of 110 participants to detect at least a 15 percentage point increase in MPR ≥95 % (from 60 % of participants with MPR ≥95 % in the NAC only comparison arm to 75 % in the combined NAC plus food or cash transfer arms), assuming 15 % loss to follow-up and alpha = 0.05. In total, 788 participants are needed for the study.

### Analysis plan

We will first conduct a modified intent-to-treat analysis including participants with at least one post-enrollment visit. We will express the relationship between treatment arm (NAC, NAC plus food assistance, and NAC plus cash transfer) and MPR ≥95 % as risk differences with 95 % confidence intervals (CI). CIs will be adjusted for multiple comparisons between the three treatment groups using Bonferroni’s adjustment and the *pwcompare* compare command in Stata 14 (College Station, Texas).

To assess Objective 1, we will compare the proportion of individuals with MPR ≥95 % among those receiving NAC plus food to those receiving NAC plus cash transfers at 6 months. Only Objective 1 will be assessed in terms of non-inferiority; all other comparisons will be tests for superiority. The proportion of participants with MPR ≥95 % in both groups and the associated two-sided 95 % CI for the difference will be estimated. Non-inferiority of NAC plus cash transfers will be defined as the lower 95 % CI of the between-group difference in the primary outcome lying above a threshold of −10 % [55, 56]. If non-inferiority is evident, we will then assess whether NAC plus cash transfers is superior to NAC plus food transfers using a Wald test with alpha = 0.05.

To assess Objective 2, if the NAC plus food and cash transfer groups are not statistically different from each other based on examination of 95 % confidence intervals, we will pool the groups together and create a binary explanatory variable indicating NAC alone or NAC plus cash or food. We will then examine the effect of receiving NAC plus food or cash assistance on ART adherence compared to NAC alone.

We will potentially conduct two secondary analyses. First, if the randomization scheme produces imbalanced baseline characteristics among study participants in each of the three groups, these covariates will be included in regression models to generate adjusted risk differences and 95 % CIs. In addition, we will conduct a per protocol analysis using data from participants who received at least a minimum exposure to the interventions and for whom MPR was available at 6 months. Thus, we will construct a regression model with the 3-level treatment group and restrict the intervention groups to individuals who received ≥1 food basket or cash transfer during the 6-month intervention period.

### Human subjects protection

The Tanzanian National Institute of Medical Research and the Committee for Protection of Human Subjects at the University of California, Berkeley approved this study.

### Study status

The study was launched in December 2013 and all sites were actively enrolling participants by February 2014. As of June 2015, 784 participants had enrolled into the study.

## Discussion

There is growing recognition that the individual and public health benefits of ART cannot be fully achieved without mitigating the detrimental consequences of food insecurity [6–8]. This study will explore several strategies to alleviate food security among ART initiates in Shinyanga, Tanzania, a resource-constrained environment with widespread food insecurity. Although a limited number of studies have demonstrated that food or cash assistance may improve adherence to treatment and/or care [13–16, 25, 27, 57], the evidence base is limited either in geographic scope (i.e., high-income countries) or in the quality of the study design. This study will build on this prior research and will use rigorous methods to examine these hypothesized relationships. The careful selection of the size and composition of the food and cash transfers will ensure that the results are generalizable to other settings in eastern and southern Africa and are relevant to ongoing discussions about how best to support PLHIV to form good adherence habits.

Specifically, the results from this study will have implications for the development of supportive strategies for PLHIV in the early stages of treatment. If the results for one of the intervention arms (NAC plus food or cash assistance) are positive, demonstrating that PLHIV who receive the intervention are more likely to be retained in care and/or have better treatment adherence, a large-scale impact evaluation to determine cost-effectiveness and examine issues of scale and sustainability may be warranted. Furthermore, if this study validates the hypothesis that increased food and/or cash can mitigate food security and improve adherence, it will support the development and evaluation of livelihood interventions targeted to AIDS-affected households which aim to increase household food and economic security through training, access to productive assets (e.g., fertilizer), or financial products (e.g., microloans) [58]. In this way, this study provides important proof-of-concept data that will inform both targeted food interventions for PLHIV as well as structural income-enhancing interventions that benefit the entire household. If neither intervention arm is successful, it will suggest that limited resources should not be spent on these models without modifications to enhance their effectiveness, which would need to be evaluated in a future study. Thus, the results from the study should influence policies that aim to mitigate food and economic insecurity and maximize adherence to treatment and care among PLHIV.

Although the interventions evaluated in this study are designed to mitigate food insecurity, they are not intended to examine the effect of nutritional quality on adherence or clinical outcomes. Similarly, the interventions are also not designed specifically to mitigate malnutrition; although they may improve nutritional status, there are other pathways through which the intervention may improve ART adherence and retention in care (e.g., such as mitigating side effects) [5]. Other studies have examined the effect of nutritional composition and quality on HIV disease progression [59–61], of which some focus on ready-to-use therapeutic food (RUTF) products and/or fortified blended foods such as corn soy blend. In this study, locally available and preferred foods were selected instead of RUTF or fortified blended products in order to improve the likelihood that the results are generalizable to other settings and also to ensure that participants in the NAC plus cash transfer arm have access to the exact same foods, if they choose to purchase them.

A potential threat to the study is that the food basket and cash transfer may be shared with others in the household and not used by the PLHIV for whom it was intended. This persistent challenge with sharing has been noted [13, 33], and consequently some have provided monthly food baskets for the entire household [13, 14]. We determined that this quantity of food would be prohibitively expensive, unsustainable, cost-prohibitive, and may be coercive in participating clinics in Shinyanga Region, where 90 % of ART initiates report moderate or severe food insecurity (data not shown). The approach in this study was to *supplement* the household food supply rather than replace it, recognizing that although sharing is a threat to the intervention’s effectiveness on ART adherence, it may benefit others in the household, including children. If we determine through patient interviews that the amount of sharing food and/or cash is significant, it provides valuable information to policymakers about the limitations of cash and in-kind transfers to improve the health of individual PLHIV.

A limitation of the study is that it will not be powered to examine heterogeneity of effect by sex or site. Data from other cash transfer studies indicate that the magnitude and durability of the effect on health outcomes may be different between men and women [62, 63], a phenomenon that this study will not be powered to address. Similarity, we will not be powered to examine differential effects by study site. Furthermore, at the conclusion of the study we will not know the exact pathways through which the intervention(s) worked, although we will explore this in an ancillary qualitative study where we will focus on how the transfers were used by beneficiaries.

This study also has significant strengths. This study focuses on food insecure PLHIV who are *at risk* of malnutrition but are not severely malnourished (BMI ≥ 16). This study population was selected in order to mitigate the effects of food insecurity before the development of undernutrition, which reduces the benefits of treatment [28–30]. In addition, the inclusion of a NAC-only arm will allow study investigators to determine whether the addition of food or cash transfers results in better adherence over and above NAC alone. This issue is highly policy relevant to a variety of settings that already conduct food and nutrition assessments among HIV-infected clients. In addition, the presence of NAC in all study arms may increase the likelihood that the intervention improves nutritional status, compared, for example, to cash transfers delivered without nutritional counseling. Previous studies have found that cash transfers’ effect on nutrition among children is enhanced with the inclusion of health-related requirements [64]. Furthermore, because we will measure the effect of the food and cash transfers distributed in months 0–6 and again 6 months after they are withdrawn, we will be able to examine durability of effect. Indeed, in every randomized study of cash transfers and ART adherence that examined durability of effect (all conducted in the U.S.), adherence returns to baseline levels once the incentives are removed, hinting at the essential role of intrinsic motivation once external rewards are discontinued (a construct from Self-Determination Theory) [25].

Perhaps the most important strength of the study is that, depending on the results, the direct comparison of food to cash transfers will provide additional support for policies favoring cash assistance or will provide rationale for the continued investment in food supplementation for PLHIV. Cash transfer programs targeting poor and vulnerable households are expanding rapidly in Africa, alongside generalized HIV epidemics [65–67]. This includes TASAF, a government-run anti-poverty program that targets vulnerable households [43]. It is increasingly recognized that programs like TASAF have “spillover” benefits on reproductive health and behaviour (e.g., contraceptive use, sexual behavior) that are not directly linked to the conditions of the transfers nor the programs’ primary anti-poverty goals, as was observed in Mexico, South Africa, and Kenya [68–74]. It may be justified to expand the reach of these programs to HIV-affected households if the results from our study demonstrate that cash transfers have significant benefits on adherence to treatment and care, nutritional status, and/or household welfare. In contrast, if we find that food support is superior to cash support, this will renew the urgency to develop cost-effective ways to increase food availability, whether through livelihood interventions or the direct provision of food. In either case, the study will yield important information to guide research and policy about how best to support PLHIV who are initiating treatment.

## Abbreviations

AMPATH: Academic model for prevention and treatment of HIV/AIDS
ARV: Antiretroviral
ART: Antiretroviral therapy
BMI: Body mass index
CI: Confidence interval
MPR: Medication possession ratio
NAC: Nutrition assessment and counseling
NACS: Nutrition assessment, counseling and support
OR: Odds ratio
PEPFAR: President’s emergency plan for AIDS relief
PLHIV: People living with HIV infection
RUTF: Ready-to-use therapeutic food
TASAF: Tanzania social action fund
VAS: Visual analog scale
WHO: World health organization

## Footnotes

1 The study was also initiated in a 4^th^ facility. However, after several months it was determined that the site was not suitable for the study due to its rural location, and participant enrollment was subsequently halted.
